# Acute kidney injury in hospitalized patients with COVID-19: a retrospective cohort

**DOI:** 10.5935/2965-2774.20230428-en

**Published:** 2023

**Authors:** Fernando Godinho Zampieri, Henrique Palomba, Fernando Augusto Bozza, Daniel C. Cubos, Thiago G Romano

**Affiliations:** 1 Intensive Care Unit, Hospital Vila Nova Star - São Paulo (SP), Brazil; 2 Instituto D’Or de Pesquisa e Ensino - Rio de Janeiro (RJ), Brazil

## TO THE EDITOR

Coronavirus disease 2019 (COVID-19) has been reported to cause acute kidney injury (AKI).^(1-4)^ Although severe acute respiratory syndrome coronavirus 2 (SARS-CoV-2) may directly harm the kidneys through endothelial and coagulation dysfunction,^(1)^ AKI in COVID-19 may also be related to additional organ dysfunctions and other host factors, including mechanical ventilation. The incidence of AKI in hospitalized COVID-19 patients has been suggested to be close to 10.6%, with AKI being strongly associated with increased mortality.^(2)^ We sought to describe the occurrence of AKI in a cohort of hospitalized patients in a private network of hospitals in Brazil during the first COVID wave (March to August 2020). Second, we assessed the interplay between the time of initiation of mechanical ventilation and the occurrence of AKI. Our initial hypothesis was that AKI would predominantly occur after the initiation of mechanical ventilation. The study was approved by the centralized ethics committee with a waiver for consent due to the retrospective nature of its analysis based on anonymized data.

**Figure 1:**
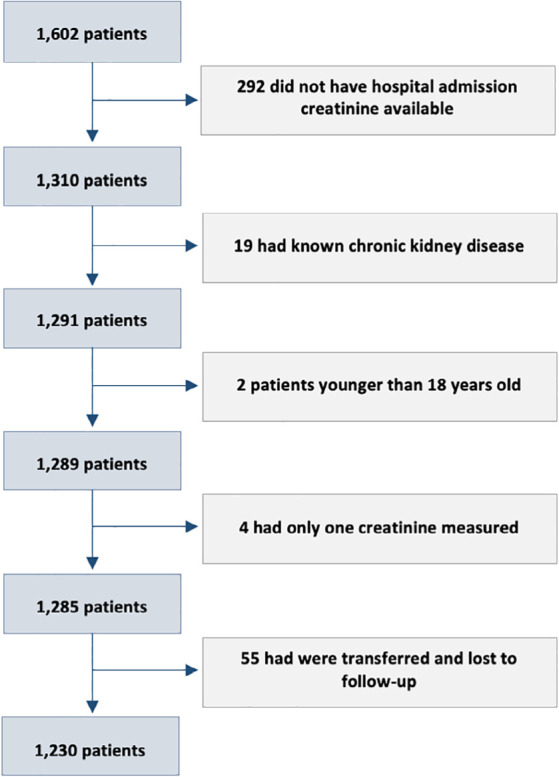
Patients selection.

We initially selected all 1,602 patients admitted to 45 hospitals in the first wave who had creatinine levels obtained at admission, who did not have a diagnosis of chronic kidney disease, who were older than 18 years old, who had at least one additional creatinine measurement, and who had known hospital outcomes (not transferred to another facility), as shown in figure 1. AKI was defined using two different definitions based on daily information collected up to 14 days after hospital admission: (1) any increase in serum creatinine above the admission creatinine of at least 0.3mg/dL or the use of kidney replacement therapy (that is, any Kidney Disease: Improving Global Outcomes - KDIGO criteria of at least one); and (2) any doubling of creatinine or use of kidney replacement therapy - KRT (that is, a KDIGO of at least 2). Hospital outcome was also collected from records. The patients’ information is shown in table 1.

**Table 1:** Patient features according to acute kidney injury

Characteristic	KDIGO ≥ 1		KDIGO ≥ 2	
	No (n = 1,047)	Yes (n = 183)	No (n = 1,126)	Yes (n = 104)
Age, median	52 (42 - 63)	60 (48 - 72)	53 (42 - 64)	62 (52 - 74)
Male sex	612 (58)	117 (64)	667 (59)	62 (60)
Urea (mg/dL)	29 (23 - 37)^*^	41 (29 - 67)†	29 (23 - 37)‡	51 (34 - 82)§
Creatinine (mg/dL)	0.93 (0.77 - 1.12)	1.04 (0.76 - 1.49)	0.94 (0.77 - 1.13)	1.20 (0.79 - 2.42)
Cancer	28 (2.7)	8 (4.4)	30 (2.7)	6 (5.8)
COPD	36 (3.4)	15 (8.2)	40 (3.6)	11 (11)
Hypertension	720 (69)	134 (73)	781 (69)	73 (70)
Noninvasive ventilation on Day 1	68 (6.5)	19 (10)	76 (6.7)	11 (11)
Mechanical ventilation on Day 1	22 (2.1)	60 (33)	32 (2.8)	50 (48)
Hospital mortality	25 (2.4)	81 (44)	33 (2.9)	73 (70)

COPD - chronic pulmonary obstructive disease.

* Missing 120 values; † missing 11 values; ‡ missing 126 values; § missing 5 values. The results are expressed as the median (interquartile range) or n (%).

**Figure 2:**
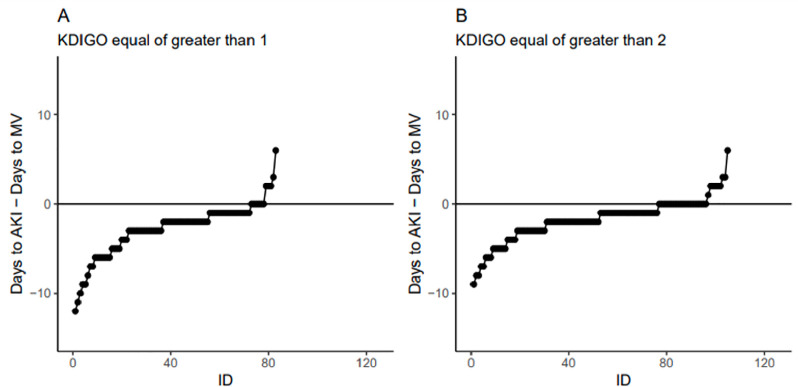
Difference in days between the day of diagnosis of acute kidney injury minus the day of the start of mechanical ventilation (y-axis) for each individual patient (x-axis) according to the acute kidney injury definition used.

A total of 1,230 patients were analyzed. Using definitions 1 and 2, AKI occurred in 183 patients (14.8%, at a median 5 days after admission, interquartile range - IQR 3 - 8 days) and 104 patients (8.4%, at a median 3 days after admission, IQR 1 - 7 days), respectively. Sixty-eight patients (5.5%) used any form of kidney replacement therapy, and 162 (13.1%) required mechanical ventilation. Acute kidney injury usually occurred after the start of mechanical ventilation (median of 2 days after, IQR 4 to 1 for definition 1, and 1 day after, IQR between 3 and 0 days for definition 2). Figure 2 shows the difference between the day of AKI diagnosis and the day of start of MV according to the AKI definitions. The use of mechanical ventilation, AKI and outcomes are shown in table 2.

**Table 2:** Raw outcomes according to mechanical ventilation and acute kidney injury

Mechanical ventilation	Acute Kidney Injury	Survival	Death
KDIGO > 1			
No	No	990 (> 99)	7 (< 1)
No	Yes	70 (99)	1 (1)
Yes	No	32 (64)	18 (36)
Yes	Yes	32 (29)	80 (71)
KDIGO > 2			
No	No	1,046 (> 99)	7 (< 1)
No	Yes	14 (93)	1 (7)
Yes	No	47 (64)	26 (36)
Yes	Yes	17 (19)	72 (81)
KRT			
No	No	1,055 (> 99)	8 (< 1)
No	Yes	5 (100)	0 (0)
Yes	No	54 (55)	45 (45)
Yes	Yes	10 (16)	53 (84)

KDIGO - Kidney Disease: Improving Global Outcomes; KRT - kidney replacement therapy; Results expressed as n (%).

AKI - acute kidney injury; MV - mechanical ventilation; KDIGO - Kidney Disease: Improving Global Outcomes.

In conclusion, AKI occurred in at least 14% of all hospitalized COVID patients during the first wave. AKI timing was strongly related to the initiation of mechanical ventilation. These findings may suggest that hemodynamic effects of mechanical ventilation and organ crosstalk may be more important than direct COVID effects in the kidney.^(5)^
